# Expression Profiling of Selected Glutathione Transferase Genes in *Zea mays* (L.) Seedlings Infested with Cereal Aphids

**DOI:** 10.1371/journal.pone.0111863

**Published:** 2014-11-03

**Authors:** Hubert Sytykiewicz, Grzegorz Chrzanowski, Paweł Czerniewicz, Iwona Sprawka, Iwona Łukasik, Sylwia Goławska, Cezary Sempruch

**Affiliations:** Siedlce University of Natural Sciences and Humanities, Department of Biochemistry and Molecular Biology, Siedlce, Poland; National Taiwan University, Taiwan

## Abstract

The purpose of this report was to evaluate the expression patterns of selected glutathione transferase genes (*gst1*, *gst18*, *gst23* and *gst24*) in the tissues of two maize (*Zea mays* L.) varieties (relatively resistant Ambrozja and susceptible Tasty Sweet) that were colonized with oligophagous bird cherry-oat aphid (*Rhopalosiphum padi* L.) or monophagous grain aphid (*Sitobion avenae* L.). Simultaneously, insect-triggered generation of superoxide anion radicals (O_2_^•−^) in infested *Z. mays* plants was monitored. Quantified parameters were measured at 1, 2, 4, 8, 24, 48 and 72 h post-initial aphid infestation (hpi) in relation to the non-infested control seedlings. Significant increases in *gst* transcript amounts were recorded in aphid-stressed plants in comparison to the control seedlings. Maximal enhancement in the expression of the *gst* genes in aphid-attacked maize plants was found at 8 hpi (*gst23*) or 24 hpi (*gst1*, *gst18* and *gst24*) compared to the control. Investigated *Z. mays* cultivars formed excessive superoxide anion radicals in response to insect treatments, and the highest overproduction of O_2_^•−^ was noted 4 or 8 h after infestation, depending on the aphid treatment and maize genotype. Importantly, the Ambrozja variety could be characterized as having more profound increments in the levels of *gst* transcript abundance and O_2_^•−^ generation in comparison with the Tasty Sweet genotype.

## Introduction

Maize (*Zea mays* L.) has increasingly emerged as a pivotal model plant species (Poaceae family, Panicoideae subfamily) that is widely used in a variety of genetic and ecotoxicological experiments [1]–[3]. During the last decade, its world production and utilization in many sectors of industrial production was substantially increased; therefore, it is important to get better insight into the complex mechanisms underlying maize tolerance towards a vast array of biotic and abiotic stressors [4]–[5]. Among the numerous insects attacking *Z. mays* plants, destructive influence of cereal aphids (Hemiptera, Aphidoidea) colonization should be underlined [6]–[8]. These phloem feeding parasites are involved in severe exploitation of the host systems, resulting in a broad range of detrimental effects, such as mechanical injuries of the stylet-penetrated tissues, local chlorosis or necrosis, deformations of organs, biomass reduction, significant disturbances of cellular homeostasis and transmission of pathogenic viruses. The harmfulness of the aphid attack is linked to the suppression of photosynthesis, diminution in chlorophyll content, intensive removal of water and photosynthates from the sieve elements [9]–[12]. Recently, there has been evidence showing that the severity of aphid-induced damages is largely associated with the composition of species-specific elicitors present in the salivary secretions injected into the host tissues [13]–[14]. Importantly, an aphid-triggered oxidative burst in tissues of host systems colonized by these hemipterans has scarcely been reported [6], [15]. On the other hand, it has been documented that cereal aphids evoked a significant decrease in ascorbate content in triticale and deterioration of the antioxidative capacity toward DPPH (1,1-diphenyl-2-picrylhydrazyl) radicals in maize plants [6], [16]. It should be noted that cellular redox imbalance in plant cells due to a chronic overproduction of various reactive oxygen species (ROS) may result in profound oxidative damages of lipids, polysaccharides, proteins and nucleic acids [15]–[16].

Cytosolic glutathione transferases (GSTs, E.C.2.5.1.18) embrace a multifunctional superfamily of enzymes participating in many physiological processes involved in plant growth and development, shoot regeneration and adaptability to adverse environmental stimuli [17]. Plant GSTs catalyze the nucleophilic substitution or addition reactions of endogenous substrates and xenobiotics with glutathione molecules, leading to the synthesis of less toxic compounds with greater solubility in water, which secondarily improves their vacuolar sequestration [18]–[19]. Additionally, glutathione transferases are involved in scavenging of excessive amounts of ROS generated in plant tissues under oxidative stress conditions, and they participate in the signal transduction pathways, cellular responses to auxins and cytokinins, as well as metabolic turnover of cinnamic acid and anthocyanins [20]–[21]. According to Dixon et al. [22], AtGSTZ1-1 from *Arabidopsis thaliana* L. possesses maleylacetone isomerase activity and participates in tyrosine degradation. Furthermore, GSTs display glutathione-peroxidase activity associated with the reduction of hydroperoxides [23]. Some authors have proposed that the activation of glutathione transferases in plants exposed to different stressors is associated with an increased ability to neutralize the lipid hydroperoxides synthesised in oxidatively damaged membranes [24]–[25]. It has been previously reported that GST isoforms overexpressed in transgenic plants markedly augment tolerance levels to herbicide treatment and oxidative stress [26]–[27]. Consistent with these observations, tau-GST from *Lycopersicon esculentum* Mill., elevated resistance to hydrogen peroxide-stimulated stress and repressed *Bax*-stimulated apoptosis in transformed yeast cells [28]. Likewise, upregulation of several plant glutathione transferases in catalase-deficient mutants were reported [29].

There are numerous studies indicating a rapid and substantial increase in the activity of various plant GST isozymes and differential regulation of *gst* genes influenced by multifarious external factors (e.g. heavy metals, herbicides, drought, low and high temperatures, UV radiation, exogenous application of chemical inducers of oxidative stress, insect infestation and fungal or viral infection) [30]–[32]. However, there is a lack of published data concerning expression profiling of the *gst* genes and superoxide anion radical (O_2_^•−^) production in the seedlings of maize varieties exposed to cereal aphid colonization. It may be assumed that mono- and oligophagous aphids differentially affect the transcriptional activity of *gst* genes and O_2_^•−^generation in tissues of maize genotypes, exhibiting diverse resistance levels to the aphid infestation. To verify this hypothesis, the relative quantification of four *gst* genes (*gst1*, *gst18*, *gst23* and *gst24*) was performed and the amount of O_2_^•−^ was measured in the seedlings of *Z. mays* Ambrozja (susceptible) and Tasty Sweet (relatively resistant) varieties infested by monophagous grain aphid (*Sitobion avenae* F.) or oligophagous bird cherry-oat aphid (*Rhopalosiphum padi* L.). The study was also aimed at assessing whether the scale of aphid-triggered changes in the levels of the analysed parameters may be dependent on the insect density.

## Methods

### Plant material

The seeds of two investigated *Z. mays* varieties (Ambrozja and Tasty Sweet) were acquired from local commercial grain suppliers: Reheza (Moszna, Poland) and PNOS S.A. (Ożarów Mazowiecki, Poland). Before performing the bioassays, intact maize seeds without any visible damages were surface sterilized as described previously [32]. Subsequently, portions of plant material (5 seeds of each cultivar per plate; four replicates) were subjected to potato dextrose agar (PDA) plate screening in order to confirm the absence of mycoflora, according to the method of Adejumo et al. [33]. Ambrozja genotype has previously been classified as relatively resistant, whereas Tasty Sweet is susceptible to the cereal aphids' infestation [6]. Maize seeds were sown in round plastic pots (10×9 cm; diameter × height) filled with general-purpose horticultural substrate and no additional fertilization was applied. Seedlings were grown in a climate chamber at 22±2°C/16±2°C (day/night) with a light intensity of 100 *µ*M m^−2^ s^−1^, a long-day photoperiod (L16: D8) and a relative humidity of 65±5%. It is important to note that only health maize seedlings of similar height were included during the experiments.

### Aphids

Wingless parthenogenetic females of *R. padi* and *S. avenae* aphids were collected from the field crops within the Siedlce district, Poland (52°09′54″N, 22°16′17″E). The authors state that no specific permissions were required for the sampling of aphids in this location, and confirm that the field studies did not involve endangered or protected species. The collected females were transferred to the seedlings of common wheat (*Triticum aestivum* L.) cv. Tonacja in the Department of Biochemistry and Molecular Biology, University of Natural Sciences and Humanities (Siedlce, Poland). New wheat seedlings were provided every week, and the aphids were reared for a year in the climate chamber under the conditions described above. Adult apterous females of the cereal aphids used in the leaf infestation experiments originated from the mother stock cultures of parthenogenetic individuals.

### Infestation experiments

Leaves of 14-day-old maize seedlings (Ambrozja and Tasty sweet cultivars) were colonized with 10, 20, 40, or 60 adult wingless females of the relevant cereal aphids (*R. padi* or *S. avenae*) per plant. The control groups of seedlings were not infested with hemipterans. The levels of relative expression of the selected *gst* genes (*gst1*, *gst18*, *gst23* and *gst24*) and O_2_^•−^ generation in *Z. mays* seedling leaves were determined 1, 2, 4, 8, 24, 48, and 72 h after initial insect infestation (hpi). Maize plants infested with aphids and the non-infested (control) plants were isolated in gauze-covered plastic cylinders (20×50 cm; diameter × height). At the end of each variant of biotests, the aphids were removed from the plants and, subsequently, the seedling leaves were excised and used immediately for further analytic procedures.

### Determination of superoxide anion radical generation in the maize seedlings

The formation of O_2_^•−^ was measured by the reduction of nitroblue tetrazolium (NBT), according to the method of Chaitanya and Naithani [34] with necessary modifications. Freshly collected *Z. mays* seedling leaves were cut into small pieces, and 0.5 g of the plant material was homogenized in 5 cm^3^ of ice-cold phosphate buffer (100 mM, pH 7.2) with 1 mM diethyldithiocarbamate (superoxide dismutase inhibitor). The homogenate was filtered through four layers of nylon mesh and centrifuged at 19 000×*g* for 20 min at 4°C. A portion of the supernatant (0.2 cm^3^) was combined with 0.8 cm^3^ of the phosphate buffer and 0.1 cm^3^ of 25 mM NBT (Sigma-Aldrich, Poland), and then, the mixture was incubated at 25°C for 5 min. Absorbance values of the sample before the incubation (*A*_0_) and after the incubation period (*A*_S_) were determined at 540 nm using an Epoch UV-Vis microplate spectrophotometer (BioTek, USA). The amount of O_2_^•−^ in *Z. mays* seedling leaves was calculated using the following formula: Δ*A*_540_ =  *A*_S_ – *A*_0_, and it was expressed as Δ*A*_540_ (min^−1^ g^−1^) fresh weight.

### Isolation of total RNA and cDNA synthesis

The insect-infested and non-infested seedling leaves of both investigated *Z. mays* genotypes were collected and homogenized immediately in liquid nitrogen by employing a sterile ceramic mortar and pestle. Total RNA was extracted with the application of *Spectrum Plant Total RNA Kit* (Sigma Aldrich, Poland) and, subsequently, trace amounts of genomic DNA were degraded using the *On-Column DNase I Digestion Set* (Sigma Aldrich, Poland). The quantitative-qualitative evaluation of the RNA samples was conducted with the use of an Epoch UV-Vis microplate spectrophotometer (BioTek, USA). High-quality RNA preparates (*A*_260/280_ >2.0; *A*_260/230_ >1.8) were exclusively accepted for the reverse-transcription reaction. Synthesis of complementary DNA (cDNA) was performed with the use of *RevertAid Premium First Strand cDNA Synthesis Kit* (Fermentas, Poland). It should be noted that the protocol scheme with oligo(dT)_18_ primers was applied. Additionally, two negative controls (NTC – no template control, and NRT – no reverse transcriptase) were included.

### Gene expression quantification

The relative expression of the target *gst* genes in foliar tissues of the aphid-infested and non-infested (control) *Z. mays* seedlings was estimated using the quantitative real-time reverse-transcription polymerase chain reaction (qRT-PCR). The glyceraldehyde-3-phosphate dehydrogenase (*gapdh*) gene was used as the internal reference [6]. Transcriptional activity of four *gst* genes (*gst1*, *gst18*, *gst23* and *gst24*) was measured with the application of TaqMan Gene Expression Assays (Life Technologies, Poland). The selection of target genes was based on their regulation under specific stress conditions (*gst1* has widely been described as a molecular marker of oxidative stress in maize tissues and *gst23* has been thought to be associated with multiple disease resistance, whereas expression of *gst18* and *gst24* genes was markedly altered under fungal infections) [19], [32]. Reference sequences and unique assay names (IDs) of the quantified *gst* transcripts are listed in Table S1. The reaction mixtures (20 mm^3^ final volume) contained 10 mm^3^ 2× TaqMan Fast Universal PCR Master Mix, 1 mm^3^ 20× TaqMan gene expression assay solution, 4 mm^3^ template (cDNA) and 5 mm^3^ RNase-free deionised water. Detection of the fluorescence signals was carried out on the StepOne Plus Real-Time PCR System equipped with StepOnePlus Software v2.3 (Life Technologies, USA). Amplification plots were obtained under the following thermal cycling conditions: initial activation of AmpliTaq Gold DNA polymerase at 95°C (20 s) and, subsequently, 40 cycles of 95°C (1 s) and 60°C (20 s). Relative gene expression was estimated according to the comparative *C*_T_ (ΔΔ*C*_T_) method [35], and the results are reported as the mean *n*-fold change ± standard deviation (SD) in the specific transcript amount of the aphid-stressed plants compared to the relevant non-infested control plants. Three biological and three technical replicates were included for each tested sample.

### Statistical analysis

The data are presented as the mean ± SD of three independent experiments. Each group of aphid-stressed and non-infested maize plants consisted of ten seedlings of a similar height. Factorial analysis of variance (ANOVA) was applied to assess the effects of four experimental indicators (maize cultivar, hemipteran species, insect abundance and aphid exposure period) as well as their interdependence. Afterwards, a post-hoc Tukey's test was performed (*p* values less than 0.05 were considered significant). Statistical analyses were carried out with the implementation of STATISTICA 10 software (StatSoft, Poland).

## Results

### Effects of cereal aphids colonization on O_2_^•−^ generation in *Z. mays* seedlings

Both *R. padi* and *S. avenae* aphids accelerated O_2_^•−^ production in the colonized Ambrozja and Tasty Sweet maize cultivars compared with the relevant control plants (Table 1, 2). Bird cherry-oat aphid infestation led to a greater increase in O_2_^•−^ amounts than grain aphid attack. For example, at 4 hpi, colonization of Tasty Sweet or Ambrozja plants with bird cherry-oat aphids at the highest density (60 per seedling) led to 65 and 209% increases in the O_2_^•−^ levels relative to the control, respectively, whereas infestation of these cultivars with the same number of *S. avenae* and insect exposure time led to 49 and 117% increases in O_2_^•−^, respectively. Ambrozja seedlings that were attacked with either aphid species were characterized by significantly higher production of O_2_^•−^ than the insect-stressed Tasty Sweet plants (Table 1, 2). The lowest initial number of both aphid species (10 per seedling) resulted in slight increments in the superoxide anion radicals content in the leaves of both maize varieties in relation to the control. Plants treated with higher numbers of aphids showed proportionally greater levels of O_2_^•−^ accumulation. Consequently, the largest differences in aphid-stimulated production of O_2_^•−^ between the two maize cultivars were observed at the highest insect density (60 per seedling). For example, *R. padi*–stressed Tasty Sweet plants generated 2–26% more O_2_^•−^ (depending on duration of aphid colonization) than seedlings attacked by *S. avenae*, whereas *R. padi*–stressed Ambrozja plants had 4–91% greater rates of O_2_^•−^ formation than *S. avenae*–infested seedlings. Additionally, slightly more superoxide anion radicals production was found in the non-infested Ambrozja seedlings than in Tasty Sweet plants (Table 1, 2). Importantly, the duration of aphid infestation had a strong influence on the generation of O_2_^•−^ in leaves of both *Z. mays* genotypes. Comparative analysis of all treatments revealed that the lowest level of O_2_^•−^ generation occurred at 1 hpi (2–9% increase, depending on the aphid infestation level) relative to the control. Maximal O_2_^•−^ formation was observed at 4 hpi in Tasty Sweet seedlings infested with 60 individuals of bird cherry-oat aphid or grain aphid, and in Ambrozja seedlings colonized with 20–60 *R. padi* or 40–60 *S. avenae* aphids per plant. For the other tested bioassay variants, the highest O_2_^•−^ generation occurred after 8 hpi compared to the non-stressed seedlings. Prolonged aphid feeding resulted in a progressive decrease in the amount of analysed ROS in comparison to maximal changes observed after 4–8 h of aphid colonization. Furthermore, factorial analysis of variance (ANOVA) revealed significant effects of the experimental indicators and their interactions on levels of O_2_^•−^ production in the maize seedlings (Table 3).

**Table 1:** Levels of O_2_^•−^ generation (Δ*A*_540_ min^−1^ g^−1^ fresh weight) in leaves of the maize seedlings colonized with *R. padi.*

Time intervals of aphid infestation (hpi)		Aphid abundance (per plant)				
		0	10	20	40	60
**Ambrozja genotype**						
0	0.47±0.03a		0.47±0.03a	0.47±0.03a	0.47±0.03a	0.47±0.03a
1	0.47±0.02b		0.47±0.02b	0.47±0.02b	0.49±0.03ab	0.51±0.04a
2	0.48±0.04b		0.48±0.04b	0.49±0.03b	0.52±0.04ab	0.58±0.03a
4	0.47±0.03d		0.50±0.04d	0.81±0.06c	1.23±0.08b	1.45±0.10a
8	0.49±0.04d		0.65±0.04c	0.70±0.05c	0.91±0.06ab	1.03±0.06a
24	0.48±0.04d		0.57±0.05c	0.62±0.05bc	0.69±0.04b	0.87±0.07a
48	0.48±0.03cd		0.55±0.04c	0.57±0.03bc	0.63±0.03b	0.81±0.06a
72	0.49±0.04c		0.53±0.03c	0.54±0.04bc	0.60±0.04ab	0.76±0.05a
**Tasty Sweet genotype**						
0	0.42±0.02a		0.42±0.02a	0.42±0.02a	0.42±0.02a	0.42±0.02a
1	0.42±0.02a		0.42±0.02a	0.42±0.02a	0.43±0.02a	0.44±0.02a
2	0.44±0.03a		0.44±0.03a	0.45±0.02a	0.47±0.03a	0.49±0.04a
4	0.43±0.02bc		0.44±0.03bc	0.47±0.03b	0.50±0.05b	0.71±0.08a
8	0.43±0.02d		0.50±0.04cd	0.53±0.05b	0.62±0.06a	0.66±0.06a
24	0.45±0.04bc		0.48±0.03b	0.51±0.04ab	0.54±0.05a	0.61±0.05a
48	0.44±0.03bc		0.45±0.04bc	0.50±0.04b	0.51±0.05ab	0.58±0.05a
72	0.43±0.03b		0.43±0.03b	0.46±0.03ab	0.48±0.03ab	0.55±0.05a

Values are the means ± standard deviation (SD) of three independent experiments (10 plants per repeat); hpi-hours post-initial insect infestation; the different letters in rows denote significant differences according to Tukey's test (P≤0.05).

**Table 2:** Levels of O_2_^•−^ generation (Δ*A*_540_ min^−1^ g^−1^ fresh weight) in leaves of the maize seedlings colonized with *S. avenae.*

Time intervals of aphid infestation (hpi)	Aphid abundance (per plant)				
	0	10	20	40	60
**Ambrozja genotype**					
0	0.47±0.03a	0.47±0.03a	0.47±0.03a	0.47±0.03a	0.47±0.03a
1	0.47±0.02a	0.47±0.02a	0.47±0.02a	0.48±0.03a	0.49±0.03a
2	0.48±0.04ab	0.48±0.04ab	0.48±0.04ab	0.50±0.04ab	0.54±0.04a
4	0.47±0.03c	0.49±0.03c	0.52±0.05c	0.88±0.07ab	1.02±0.08a
8	0.49±0.04c	0.60±0.04b	0.67±0.05b	0.65±0.04b	0.85±0.06a
24	0.48±0.04b	0.54±0.03b	0.63±0.05ab	0.62±0.05ab	0.76±0.05a
48	0.48±0.03bc	0.52±0.04b	0.59±0.03ab	0.59±0.03ab	0.68±0.04a
72	0.49±0.04bc	0.51±0.03b	0.54±0.04b	0.55±0.03b	0.63±0.05a
**Tasty Sweet genotype**					
0	0.42±0.02a	0.42±0.02a	0.42±0.02a	0.42±0.02a	0.42±0.02a
1	0.42±0.02a	0.42±0.02a	0.42±0.02a	0.43±0.02a	0.43±0.03a
2	0.44±0.03a	0.44±0.03a	0.44±0.03a	0.45±0.02a	0.48±0.04a
4	0.43±0.02b	0.45±0.03b	0.45±0.02b	0.47±0.03b	0.64±0.05a
8	0.43±0.02b	0.48±0.03ab	0.51±0.05a	0.58±0.04a	0.55±0.04a
24	0.45±0.04ab	0.47±0.02ab	0.49±0.04a	0.51±0.04a	0.52±0.05a
48	0.44±0.03ab	0.44±0.03ab	0.47±0.03a	0.49±0.03a	0.50±0.04a
72	0.43±0.03ab	0.43±0.04ab	0.44±0.03ab	0.46±0.02a	0.48±0.03a

Values are means ± standard deviation (SD) of three independent experiments (10 plants per repeat); hpi-hours post-initial insect infestation; different letters in rows denote significant differences according to Tukey's test (P≤0.05).

**Table 3:** Factorial ANOVA results for tested indicators (*Z. mays* cultivar, hemipteran species, insect abundance and aphid exposure period) and interdependence between these parameters affecting O_2_^•−^ formation in the maize seedlings.

Tested factors and interactions	Df	*F*	*p*
Maize cultivar (C)	1	175.2	≤0.001
Hemipteran species (S)	2	87.9	≤0.001
Insect abundance (A)	3	68.2	≤0.001
Aphid exposure period (EP)	7	52.7	≤0.001
S × C	2	19.6	≤0.001
S × A	6	14.5	≤0.001
C × A	3	27.1	≤0.001
S × EP	14	14.9	≤0.001
C × EP	7	18.5	≤0.001
A × EP	21	10.6	≤0.001
S × C × A	6	12.4	≤0.001
S × C × EP	14	9.9	≤0.001
S × A × EP	42	8.7	≤0.001
C × A × EP	21	4.9	≤0.001
S × C × A × EP	42	3.7	≤0.008

Df-degrees of freedom; *p*-values less than 0.05 were considered significant; F-ratio is defined as the variance between samples/the variance within samples.

### Transcriptional activity of *gst1* gene in the aphid-stressed maize seedlings

**Figure 1:**
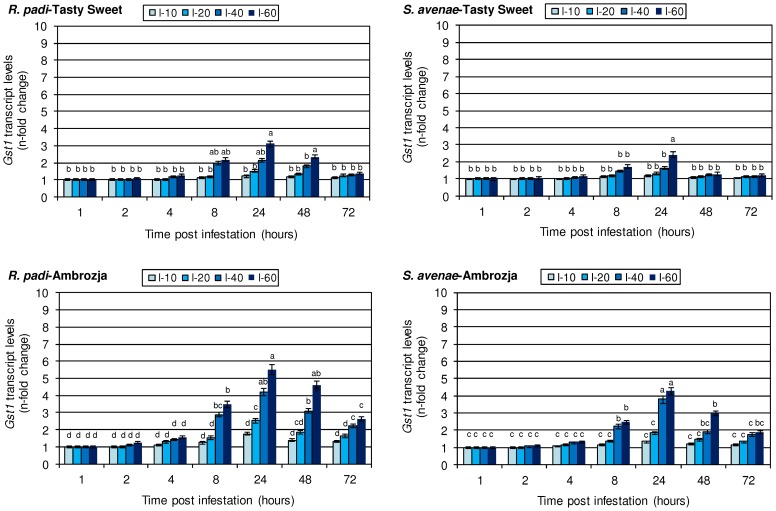
Influence of the tested cereal aphids on *gst1* gene expression in the seedlings of Ambrozja and Tasty Sweet maize cultivars. Values signify the mean *n*-fold changes in the *gst1* transcript abundance in the aphid-stressed *Z. mays* plants in comparison with the non-infested group of seedlings. Error bars represent the standard deviation (± SD). For each maize-aphid treatment, three independent biological replicates were accomplished. The obtained gene expression data were normalized to the *gapdh* gene. The different letters above the SD bars designate significant differences among compared plants at P≤0.05 based on the Tukey's test. I-10, I-20, I-40 and I-60 are the levels of aphid infestation (10, 20, 40 and 60 insects per plant, accordingly).

The conducted biotests demonstrated that short-term feeding of the examined cereal aphids (*R. padi* or *S. avenae*) did not influence the amount of *gst1* mRNA transcript in the seedlings of Ambrozja and Tasty Sweet maize genotypes (Figure 1). Two hours after initial infestation, the low abundance of aphids (10–20 individuals per plant) did not alter the gene expression, but a higher number of insects (40–60 per seedlings) stimulated a slight increment in transcriptional activity of the target gene (from 5% increase in Tasty Sweet plants colonized with 60 *S. avenae* to a 26% increase in Ambrozja plants infested with the same number of *R. padi* aphids). After 4 and 8 hpi, the levels of *gst1* transcript gradually enhanced in both maize varieties colonized with the tested aphid species, with the exception of two aphid treatments (10 and 20 insects per plant) at 4 hpi when there were no changes in the relative expression of the analysed gene in Tasty Sweet genotype. The highest accumulation of the *gst1* transcript amount in the aphid-infested maize seedlings of both *Z. mays* genotypes occurred at 24 hpi and 60 aphids per plant (4.3–5.5-fold elevations in Ambrozja, and 2.4–3.1-fold increases in Tasty Sweet seedlings, depending on the aphid species). However, extended insect colonization (48–72 hpi) resulted in a gradually lower gene expression in relation to the levels recorded at 24 hpi. Generally, *R. padi* infestation led to a more profound increase in the transcriptional activity of the *gst1* gene in comparison with *S. avenae* (e.g. 120% higher increase in Ambrozja and 69% increment in Tasty Sweet plants, at 24 hpi and 60 aphids per plant). The results of factorial ANOVA confirmed a significant influence of the analysed indicators and their interactions on expression of the *gst1* gene in the maize seedlings (Table 4).

**Table 4:** Factorial ANOVA results for tested indicators (*Z. mays* cultivar, hemipteran species, insect abundance and aphid exposure period) and interactions between these parameters affecting *gst1* and *gst18* transcript amounts in the maize seedlings.

Tested factors and interactions	Df	*F*	*p*	*F*	*p*
		*gst1* gene		*gst18* gene	
Maize cultivar (C)	1	986.8	≤0.001	916.9	≤0.001
Hemipteran species (S)	2	852.4	≤0.001	1645.2	≤0.001
Insect abundance (A)	3	1447.0	≤0.001	1362.5	≤0.001
Aphid exposure period (EP)	7	1078.3	≤0.001	1573.8	≤0.001
S × C	2	561.4	≤0.001	965.2	≤0.001
S × A	6	374.2	≤0.001	1258.9	≤0.001
C × A	3	309.2	≤0.001	724.5	≤0.001
S × EP	14	407.4	≤0.001	1419.8	≤0.001
C × EP	7	275.1	≤0.001	1160.4	≤0.001
A × EP	21	223.2	≤0.001	583.9	≤0.001
S × C × A	6	79.5	≤0.001	185.3	≤0.001
S × C × EP	14	77.8	≤0.001	306.0	≤0.001
S × A × EP	42	58.0	≤0.001	163.2	≤0.001
C × A × EP	21	45.2	≤0.001	142.5	≤0.001
S × C × A × EP	42	13.8	≤0.001	40.5	≤0.001

Df-degrees of freedom; *p*-values less than 0.05 were considered significant; F-ratio is defined as the variance between samples/the variance within samples.

### Amount of *gst18* transcript in the insect-injured *Z. mays* seedlings

**Figure 2:**
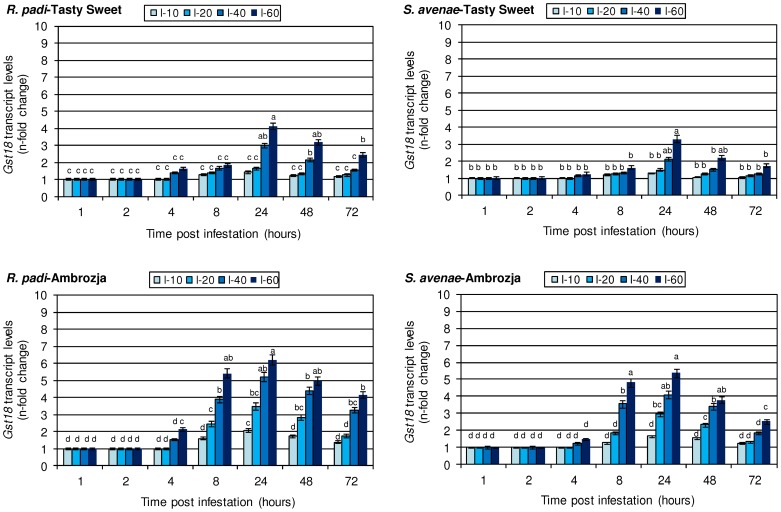
Influence of the tested cereal aphids on *gst18* gene expression in the seedlings of Ambrozja and Tasty Sweet maize cultivars. Values signify the mean *n*-fold changes in the *gst18* transcript abundance in the aphid-stressed *Z. mays* plants in comparison with the non-infested group of seedlings. Error bars represent the standard deviation (± SD). For each maize-aphid treatment, three independent biological replicates were accomplished. The obtained gene expression data were normalized to the *gapdh* gene. The different letters above the SD bars designate significant differences among compared plants at P≤0.05 based on the Tukey's test. I-10, I-20, I-40 and I-60 are the levels of aphid infestation (10, 20, 40 and 60 insects per plant, accordingly).

The performed analyses revealed that the transcriptional activity of the *gst18* gene in tissues of both maize cultivars remained unaffected after 1 or 2 h of aphid colonization (Figure 2). The 4 h infestation with a higher density of insects (40–60 per seedling) resulted in slightly enhanced levels of gene expression (16–112% increment), whereas a lower abundance (10–20 aphids per plant) did not evoke any alternations compared to the control. Eight hours after the initial infestation, the transcriptional activity of the *gst18* gene in seedlings of the investigated *Z. mays* cultivars gradually increased in proportion to the number of hemipterans per plant (21–82% increase in Tasty Sweet and 27–440% increase in Ambrozja variety). It is important to note, that the highest stimulation of target gene expression occurred at 24 hpi. Colonization of maize plants with *R. padi* aphids at this time point led to 1.4–4.1-fold and 2.1–6.2-fold elevations in the transcript abundance in Tasty Sweet and Ambrozja seedlings, accordingly, whereas the grain aphid attack resulted in 1.3–3.4-fold and 1.7–5.5-fold increases in the corresponding maize cultivars. During the next two periods of aphid infestation the scale of upregulation of the *gst18* gene in both maize genotypes was less pronounced (1.2–4.9-fold increments at 48 hpi; 1.1–4.2-fold elevations at 72 hpi, depending on the aphid treatments). Importantly, *R. padi*-colonized maize plants were characterized with greater amounts of the target transcript (20–97% Tasty Sweet and 61–163% Ambrozja) in relation to *S. avenae*-attacked seedlings. Furthermore, it was evidenced that the aphid-infested Ambrozja plants responded with much greater increases in the *gst18* gene expression compared to the infested Tasty Sweet genotype (e.g. 50–258% greater increments at 60 insects per plant). Statistical analysis confirmed the considerable impact of tested parameters and their interrelation on expression of the analysed gene in the investigated *Z. mays* plants (Table 4).

### Relative expression of *gst23* gene in maize plants colonized with cereal aphids

**Figure 3:**
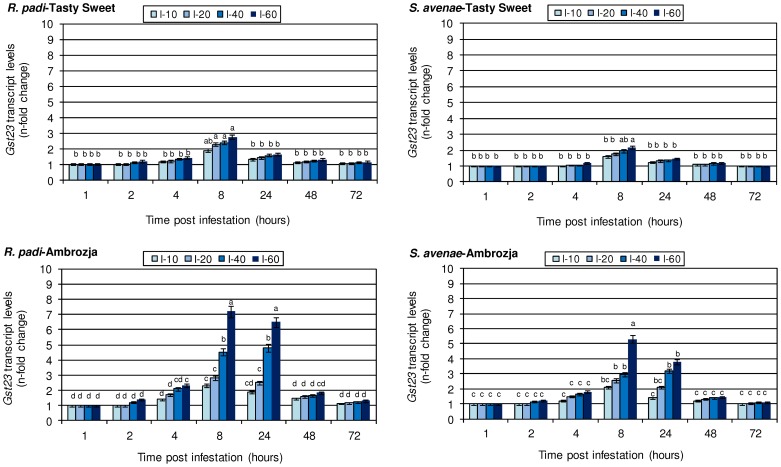
Influence of the tested cereal aphids on *gst23* gene expression in the seedlings of Ambrozja and Tasty Sweet maize cultivars. Values signify the mean *n*-fold changes in the *gst23* transcript abundance in the aphid-stressed *Z. mays* plants in comparison with the non-infested group of seedlings. Error bars represent the standard deviation (± SD). For each maize-aphid treatment, three independent biological replicates were accomplished. The obtained gene expression data were normalized to the *gapdh* gene. The different letters above the SD bars designate significant differences among compared plants at P≤0.05 based on the Tukey's test. I-10, I-20, I-40 and I-60 are the levels of aphid infestation (10, 20, 40 and 60 insects per plant, accordingly).

Results concerning the expression levels of the *gst23* gene in the aphid-infested seedlings of *Z. mays* are depicted in figure 3. It has been found that feeding *S. avenae* or *R. padi* for 1 h did not evoke any disturbances in the transcriptional activity of the target gene in tissues of the investigated maize cultivars. Insect feeding for 2 h did not result in any changes in the *gst1* gene expression in *S. avenae*-infested Tasty Sweet plants, regardless of the number of aphids per plant. Likewise, the tested cereal aphids (10–20 insects per seedling) did not affect the transcriptional activity of the analysed gene in both maize genotypes (Ambrozja or Tasty Sweet). However, higher numbers of aphids (40–60 per plant) led to an elevation in the *gst23* transcript abundance, ranging from 10% in Tasty Sweet plants colonized by 40 *R. padi* aphids to 42% increase in Ambrozja seedlings infested by 60 insects per plant. Further extension of colonization period (4 hpi) resulted in a continuous increase (3–132%) in *gst23* gene expression in the maize tissues compared with the relevant control plants. The maximal induction of the target gene in aphid-attacked maize plants occurred at 8 hpi. At this time point, 10–60 *R. padi* per plant evoked 1.9–2.8-fold and 2.3–7.2-fold increases in the levels of transcript accumulation in Tasty Sweet and Ambrozja plants, respectively. Infestation with *S. avenae* (10–60 aphids per seedling) caused 1.6–2.2-fold and 2.1–5.3-fold elevations in Tasty Sweet and Ambrozja varieties, respectively. Prolonged exposure to aphids (24–72 hpi) could be linked to a progressively lower upregulation of the *gst23* gene in comparison with the changes observed at 8 hpi. Interestingly, long-term colonization (72 hpi) by the grain aphid did not influence the analysed transcript amount in Tasty Sweet plants in relation to the relative non-infested seedlings. Comparative analyses revealed that the bird cherry-oat aphid caused a more noticeable augmentation of *gst23* gene expression (11–170%, depending on the aphid treatment and maize cultivar) in comparison with *S. avenae* aphids. Moreover, elevation of the transcriptional activity of the *gst23* gene in maize plants occurred in parallel with increasing aphid densities per plant. A markedly higher transcript amount was found in the insect-stressed Ambrozja seedlings compared to Tasty Sweet plants. For example, after 8 h infestation, 60 *R. padi* aphids stimulated 2.8- and 7.2-fold increments in Tasty Sweet and Ambrozja plants, respectively, whereas feeding the same number of *S. avenae* individuals led to 2.2- and 5.3-fold increases in Tasty Sweet and Ambrozja varieties, respectively. The results of factorial ANOVA analysis proved that there was a significant impact of the investigated parameters and their interdependence on the transcriptional activity of the *gst23* gene in the maize seedlings (Table 5).

**Table 5:** The factorial analysis of variance of tested indicators (*Z. mays* cultivar, hemipteran species, insect abundance and aphid exposure period) and interactions between these parameters affecting *gst23* and *gst24* transcript amounts in the maize seedlings.

Tested factors and interactions	Df	*F*	*p*	*F*	*p*
		*gst23* gene		*gst24 gene*	
Maize cultivar (C)	1	1142.5	≤0.001	573.2	≤0.001
Hemipteran species (S)	2	748.3	≤0.001	1229.2	≤0.001
Insect abundance (A)	3	1325.2	≤0.001	495.5	≤0.001
Aphid exposure period (EP)	7	1409.8	≤0.001	1050.9	≤0.001
S × C	2	721.4	≤0.001	147.6	≤0.001
S × A	6	438.9	≤0.001	120.8	≤0.001
C × A	3	166.3	≤0.001	29.5	≤0.001
S × EP	14	844.6	≤0.001	275.0	≤0.001
C × EP	7	275.0	≤0.001	50.6	≤0.001
A × EP	21	196.5	≤0.001	47.3	≤0.001
S × C × A	6	45.9	≤0.001	16.7	≤0.001
S × C × EP	14	69.4	≤0.001	7.4	≤0.004
S × A × EP	42	53.1	≤0.001	12.8	≤0.001
C × A × EP	21	27.7	≤0.001	3.6	≤0.006
S × C × A × EP	42	10.5	≤0.001	1.5	≤0.017

Df-degrees of freedom; *p*-values less than 0.05 were considered significant; F-ratio is defined as the variance between samples/the variance within samples.

### Abundance of *gst24* transcript in *Z. mays* seedlings infested with the cereal aphids

**Figure 4:**
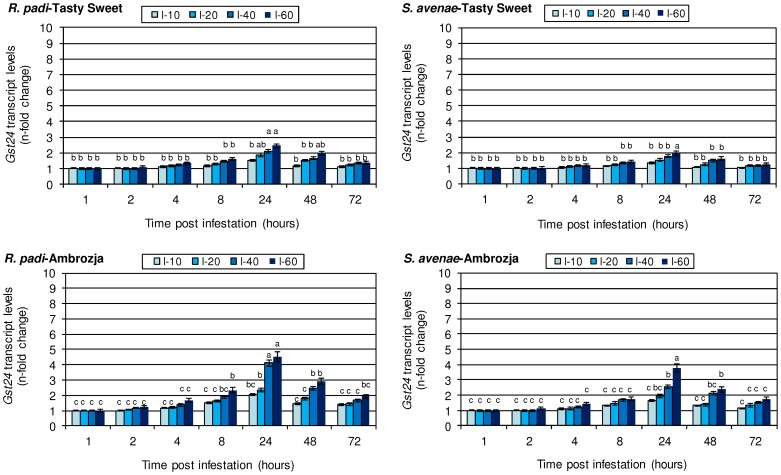
Influence of the tested cereal aphids on *gst24* gene expression in the seedlings of Ambrozja and Tasty Sweet maize cultivars. Values signify the mean *n*-fold changes in the *gst24* transcript abundance in the aphid-stressed *Z. mays* plants in comparison with the non-infested group of seedlings. Error bars represent the standard deviation (± SD). For each maize-aphid treatment, three independent biological replicates were accomplished. The obtained gene expression data were normalized to the *gapdh* gene. The different letters above the SD bars designate significant differences among compared plants at P≤0.05 based on the Tukey's test. I-10, I-20, I-40 and I-60 are the levels of aphid infestation (10, 20, 40 and 60 insects per plant, accordingly).

Relative expression data of the *gst24* gene in aphid-colonized maize seedlings are presented in figure 4. Transcriptional activity of the target gene in tissues of both tested *Z. mays* cultivars infested with *R. padi* or *S. avenae* remained at the same levels after 1 hpi, when compared to the respective control seedlings. In maize plants exposed to insect infestation for 2 h, only subtle accumulation of the *gst24* transcript was recorded (3–10% increase in Tasty Sweet seedlings, and 6–24% elevation in Ambrozja plants). Prolonged aphid colonization (4–8 hpi) was associated with a steady enhancement in the expression of the analysed gene from 5% elevation in *S. avenae*-infested Tasty Sweet plants to 133% increment in *R. padi*-attacked Ambrozja seedlings, compared to the controls. The highest enhancement in the transcript amount for tissues of the aphid-infested maize plants was found at 24 hpi (e.g. 60 *R. padi* aphids influenced 2.5-fold and 4.5-fold increases in Tasty Sweet and Ambrozja seedlings, respectively, whereas the same abundance of *S. avenae* affected 2.0- and 3.8-fold increments in the relevant maize genotypes). It should be emphasized that insect infestation for 48 and 72 h resulted in a gradually decreasing upregulation of *gst24* gene expression in *Z. mays* seedlings of the investigated cultivars in relation to the changes demonstrated after 24 h. Furthermore, the aphid-attacked Ambrozja plants responded to a higher elevation in the transcriptional activity of the target gene when compared with Tasty Sweet variety (e.g. 12–205% larger increase at the highest level of aphid infestation). It was additionally demonstrated that there was a higher abundance of the target mRNA transcript in *R. padi*-infested maize cultivars in comparison with *S. avenae*-stressed seedlings. It is important to underline that the scale of alternations in the gene expression was proportional to densities of the tested hemipterans on the seedlings of the investigated maize varieties. The maximal abundance of bird cherry-oat aphids (60 insects per plant) led to 7–50% and 9–71% higher increments of *gst24* gene expression in the Tasty Sweet and Ambrozja plants, respectively, relative to the number of grain aphids. The statistical analysis evidenced significant effects of the tested variables and their interconnections in terms of *gst24* gene expression in *Z. mays* plants (Table 5).

## Discussion

Monophagous *Sitobion avenae* F. (grain aphid) and oligophagous *Rhopalosiphum padi* L. (bird cherry-oat aphid) become serious pest species building up numerous colonies on many maize varieties grown in Poland, especially during warm and moist vegetative seasons [36]–[38]. Despite many research groups conducting extensive studies on the complex plant-aphid interactions, the participation of these hemipterans in the generation of oxidative stress and the functioning of the antioxidant defence network in the host systems still remain to be unraveled. To the best of our knowledge, this is the first report evaluating the impact of *R. padi* or *S. avenae* infestations on the expression profiles of the four genes encoding glutathione transferase isozymes (GSTF1, GST18, GST23 and GST24), as well as the levels of superoxide anion radical generation in the seedlings of susceptible (Tasty Sweet) and relatively resistant (Ambrozja) maize genotypes.

Aphid salivary glands produce a battery of hydrolytic enzymes that participate in the cleavage of primary and secondary cell walls, plasma membranes, and a variety of intracellular compounds. Additionally, salivary secretions of these hemipterans contain various elicitors, metabolic regulators, and phytotoxic constituents that trigger cascades of local and/or systemic defensive reactions as well as the processes of premature senescing, apoptosis, or necrosis within the colonized plant systems [39]–[41]. Studies have documented that proteinaceous effectors (Mp10 and Mp42) from *M. persicae* are capable of enhancing the defence systems in *Nicotiana benthamiana* Dom. plants, whereas two elicitors of *Macrosiphum euphorbiae* Thom., Me10 and Me23, possess the ability to suppress the host reactions in order to facilitate prolonged phloem feeding [13]-[14]. Aphid saliva infiltration and profound ultrastructural damages induced by insect mouthparts in the host tissues may be linked to excessive ROS release in the attacked organs. Superoxide anion radical is one of the major and most deleterious reactive oxygen species generated in plant cells both in the normal physiological state and in response to adverse environmental stimuli. It was found that the seedlings of both maize varieties colonized with *R. padi* or *S. avenae* aphids responded an early overproduction of O_2_^•–^ in comparison to the non-stressed control. The maximal enhancement in the superoxide anion radical generation in *Z. mays* seedlings was noted after 4–8 h of aphid feeding. Interestingly, a more marked elevation in O_2_^•−^ amounts occurred in the seedlings of Ambrozja (relatively resistant) plants in relation to Tasty Sweet (susceptible) cultivar. These observations are coherent with the results obtained by Mai et al. [15] who ascertained that *Pisum sativum* L. plants infested with the pea aphid (*Acyrthosiphon pisum* Harr.) possessed substantially higher amounts of O_2_^•−^ relative to the insect-free control. Furthermore, the most significant increase in excessive O_2_^•^− formation was found at the highest infestation level (30 aphids per seedling). According to these authors, the prolonged aphid feeding resulted in the progressive increase in O_2_^•−^ levels within the attacked plants (e.g. 1.46- and 1.81-fold increments in relation to the reference plants at 24 and 96 hpi, accordingly). Moreover, it was reported that Russian wheat aphid (*Diuraphis noxia* Mordv.) markedly augmented the biosynthesis of hydrogen peroxide in resistant wheat plants in relation to the aphid-susceptible line. The oxidative burst in plants is associated with a dramatic increase in superoxide anion radicals' production at early stages of the exposure to various biotic stressing factors [28]. This phenomenon is linked with subsequent oxidative wave passing throughout plant tissues, leading to triggering the defence networks in the hosts, on the one hand, and possible suppression of the growth and development of herbivorous insects, on the other hand [42]. In order to overcome the excessive accumulation of this highly reactive and cytotoxic ROS form, the superoxide anion radicals are converted in the dismutation reaction to molecular oxygen (O_2_) and less toxic hydrogen peroxide (H_2_O_2_) [6], [15]–[16], [39]. Furthermore, we revealed very slight changes in superoxide anion radicals content in non-infested maize seedlings of both tested cultivars with duration of experimental time, but the recorded differences were not statistically significant. It is probable that isolation of *Z. mays* seedlings with the cover gauze could cause a minor mechanical stress influencing negligible fluctuations in O_2_^•−^ amount.

Plants have developed a number of defence mechanisms that are involved with protecting the cells from the detrimental impact of exaggerated ROS formation in response to a variety of abiotic and biotic stresses [43]–[46]. Until now, it has been identified at least 42 genes encoding diverse isozymes of glutathione transferase in maize [47]. In recent years, an important role of cytosolic GSTs in the alleviation of oxidative stress in plant tissues has been increasingly described [48]–[52]. The GSTs predominantly occur as homo- or heterodimers, with subunits of 23–30 kDa [53]. It should be underlined that among diverse groups of GST isozymes, only Tau and Phi classes are plant specific [50]. The performed molecular studies revealed that the cereal aphid infestations led to significant increases in the relative expression of analysed *gst* genes (*gst1*, *gst18*, *gst23* and *gst24*) in the seedling leaves of both *Z. mays* genotypes, exhibiting distinct susceptibility levels to the insect colonization. Time-course analysis revealed that the target genes encoding the relevant GST isoenzymes (GSTF1, GST18, GST23 and GST24) were maximally upregulated at different aphid exposure periods (*gst23* at 8 hpi; *gst1*, *gst18*, and *gst24* genes at 24 hpi). Interestingly, the bird cherry-oat aphid infestation caused more considerable increments in the amounts of all tested *gst* transcripts in the maize plants compared to grain aphid feeding. Additionally, relatively resistant Ambrozja plants that were attacked by the cereal aphids were characterized with a higher stimulation of the transcriptional activity of the *gst* genes in relation to the susceptible Tasty Sweet plants. There have been limited reports published evidencing aphid-stimulated transcriptional reprogramming in the attacked host plants [54]–[58]. Microarray experiments performed by Kuśnierczyk and co-workers revealed that feeding of *M. persicae* or *Brevicoryne brassicae* L. for 72 h led to significant alternations in the transcriptional activity of 13 *gst* genes in 22–30-day-old plants of three tested *Arabidopsis thaliana* ecotypes (Landsberg *erecta*/L*er*/, Cape Verde Islands/Cvi/, and Wassilewskija/Ws/) [54]. The aphid colonization (8–12 insects per leaf) resulted in the overexpression of most analysed *gst* genes in the plants representing the tested ecotypes when compared to the non-stressed control. The opposite tendency was identified in the expression patterns of GSTU18 and GSTU20 transcripts (0.23–1.60-fold and 0.26–1.89-fold down regulation, respectively) in the investigated ecotypes in relation to the insect-free plants. Additionally, these authors demonstrated upregulation of the glutathione-conjugate transporter (MRP4) in the aphid-injured Cape Verde Islands and Wassilewskija plants. Another infestation experiments conducted by Kuśnierczyk et al. revealed that 21–25-day-old *A. thaliana* plants (Landsberg *erecta*/L*er/*ecotype) infested with *B. brassicae* (4 aphids per leaf) were characterized with an early enhancement (at 6 hpi) of the amount of ATGST6, ATGST7 and ATGST10 transcripts relative to the control. Furthermore, prolonged aphid colonization (48 hpi) led to the strong upregulation of four glutathione transferase (ATGSTU3, ATGSTU10, ATGSTU11, ATGSTL1) genes, as well as increases in the transcript amounts of two glutathione *S*-conjugate transporters (MRP3 and MRP4) [55]. Similarly, Moran et al. elucidated that infestation of *A. thaliana* with *M. persicae* aphids for 72 h resulted in 2.9-fold- and 4.8-fold elevations in the expression of *gst1* and *gst11* genes, respectively, compared to the non-treated control [56]. Stotz et al. also ascertained that the diamondback moth (*Plutella xylostella* L.), feeding on the rosette leaves of wild-type *A. thaliana* plants, influenced profound increments in the expression of *gst2* and *gst6* genes compared to the insect-free control [57]. Likewise, Bandopadhyay and co-workers evidenced that the transcriptional activity of genes encoding various glutathione transferase isoforms may vary significantly depending on the duration of the aphid exposure period [58]. According to the cited authors, *Rorippa indica* L. plants infested with mustard aphids (*Lipaphis erysimi*/L./Kalt.) responded with a 2.5-fold elevation in the transcriptional activity of the *AT1G78370* gene (glutathione transferase AtTAU20) at 12 hpi, but a dissimilar trend occurred when the insect colonization was extended to 48 hpi. Furthermore, it should be noted that other biotic stressors, such as pathogenic fungi or microorganisms are able to trigger notable modifications in the expression patterns of several *gst* genes within the hosts. For example, it has been elucidated that maize plants infected with *Ustilago maydis* possessed an increased transcriptional activity of seven transferase glutathione genes (*gst15*, *gst18*, *gst20*, *gst24*, *gst25*, *gst30*, and *gst36*) after 12 h post-fungal inoculation [59]. The upregulation levels ranged from a 3.1-fold increase of the *gst18* gene expression to a 108-fold increment in the *gst30* transcript abundance compared to non-treated plants. Microarray data achieved by Luo et al. revealed that the expression of transferase glutathione genes in maize kernels of aflatoxin-resistant (Eyl25) and aflatoxin-susceptible (Eyl31) lines differentially responded 72 h after inoculation with *Aspergillus flavus* [60]. Some authors have suggested that the induction of *gst* genes is involved in limiting the adverse effects of oxidative stress within plant tissues, including the reduction of cell death events occurring as a result of the hypersensitive reactions [17], [59]–[60].

In the present study, *R. padi* infestation contributed to a substantially greater upregulation of the analysed *gst* genes and to the increases in the O_2_^•−^ generation in seedlings of both Ambrozja and Tasty Sweet genotypes in comparison to grain aphid feeding. Oligophagous bird cherry-oat aphids alternate the host plants between members of the *Prunus* genus (winter hosts) and a broad set of *Poaceae* species (summer hosts), whereas the life cycle of monophagous *S. avenae* is associated with numerous grasses and cereals [10]–[11], [61]. Greater diversity of plant systems colonized with *R. padi* indicates a higher adaptation of this hemipteran species to the chemical composition of the hosts. Conceivable sources of distinct biochemical and molecular effects in aphid-infested maize plants may be caused by differences in the insect salivary compounds and specific routes of stylet insertion throughout the plant tissues. It has been reported that salivary secretions of the bird cherry-oat aphid contain a wide spectrum of biocatalysts, which are responsible for the hydrolysis of structural macromolecules in the primary and secondary cell walls, and plasmalemma [62]–[63]. Furthermore, microscopic observations conducted by some researchers have documented additional profound injuries within the mesophyll cells of both winter and summer hosts, whereas *S. avenae* infestation resulted in a much lower range of ultrastructural damages, and they displayed a typical intercellular mode of mouthparts passage within the winter wheat Sakva plants [64]–[65]. According to Urbańska et al., the bird cherry-oat aphid has evolved an adaptive enzymatic mechanism that allows detoxification of harmful cyanogenic constituents present in the leaves of primary hosts [66]. Łukasik et al. provided valuable findings, indicating that *R. padi* feeding caused greater depletion in the content of ascorbate and greater stimulation of ascorbate peroxidase activity in the triticale seedlings compared to *S. avenae* aphids [16]. Similarly, Sytykiewicz revealed that bird cherry-oat aphid infestation of maize plants evoked a more significant decrease in the total antioxidant capacity towards the DPPH (1,1-diphenyl-2-picrylhydrazyl) radical in relation to grain aphid colonization [6]. It may be assumed that a decreased efficacy of DPPH radical scavenging activity in aphid-infested maize plants might be associated with a continuous pressure of biotic stressing factor (aphid colonization) that triggered the oxidative stress in the host systems. It is particularly evident when massive and/or prolonged aphid infestation occurred. It is likely that the pool of available antioxidants under stressful conditions significantly depressed the total antioxidative capacity of extracts derived from the infested maize seedlings when compared to the control. On the other hand, lower contents of ascorbate and glutathione were evidenced, as well as higher levels of ascorbate peroxidase and glutathione transferase activities in tissues of the bird cherry-oat aphid, in comparison with *S. avenae* individuals, which proves that there are significant differences in the functioning of the antioxidative machinery within these cereal aphids [12], [67].

This report provides new insight into the molecular basis of highly complex antioxidative responses of the model maize plants colonized with cereal aphids. It was demonstrated, there is differential regulation of four *gst* genes, encoding various isoforms of glutathione transferase in the insect-challenged seedling leaves of *Z. mays*, representing high and low susceptibility to the aphid colonization. The obtained results revealed insect-triggering oxidative stress and the crucial role of glutathione transferases in constituting complex defence reactions in the attacked host systems. In order to gain a better understanding of the elicitation of the plant defence reactions which occur at the early stages of aphid infestation in maize plants, the extended molecular analyses comprising transcriptome-wide screening of other aphid-regulated genes, as well as identification of low molecular and regulatory RNA molecules (e.g. miRNA) and assessing their gene expression profiles should be performed.

## Supporting Information

Table S1 **The set of** ***Z. mays*** **glutathione transferase genes analysed with the application of** ***TaqMan Gene Expression Assays*** **^#^.** ^#^ *TaqMan Gene Expression Assays* used in the performed experiments were developed and supplied by Life Technologies (Poland).
(DOC)
