# Supplementary material for: Expression Profiling of Selected Glutathione Transferase Genes in Zea mays (L.) Seedlings Infested with Cereal Aphids
Source: PLoS One. 2014 Nov 3;9(11):e111863. doi: 10.1371/journal.pone.0111863 (PMC4218852; doi:10.1371/journal.pone.0111863)
Supplement: Table S1 — The set of Z. mays glutathione transferase genes analysed with the application of TaqMan Gene Expression Assays #. # TaqMan Gene Expression Assays used in the performed experiments were developed and supplied by Life Technologies (Poland). (DOC) [file pone.0111863.s001.doc]

**Supporting Information Table S1.**

**The set of *Z. mays* glutathione transferase genes analysed with the application of *TaqMan*® *Gene Expression Assays*^#^.**

| **Target genes** | ***GenBank* reference sequences** | **Assay identification number** | **Encoded isoenzymes** |
| --- | --- | --- | --- |
| *gst1* | NM_001111942.1 | Zm04059139_m1 | GSTF1  (Phi class) |
| *gst18* | NM_001111514.1 | Zm04051971_gH | GST18  (Zeta class) |
| *gst23* | NM_001111524.1 | Zm04057685_g1 | GST23  (Tau class) |
| *gst24* | NM_001111518.1 | Zm04051982_gH | GST24  (Tau class) |
